# Novel Technique of Dual Chamber Pacing Through Mini-thoracotomy and Transatrial Endocardial Active Fixation Lead Insertion for Epicardial Pacing Lead Malfunction

**DOI:** 10.1016/s0972-6292(16)30668-4

**Published:** 2013-09-01

**Authors:** Kothandam Sivakumar, Robert Coelho

**Affiliations:** Department of Pediatric Cardiology, MIOT Hospital, Chennai, India

**Keywords:** Peratrial pacing, Permanent pacing, Diaphragmatic pacing, Endocardial screw-in pacing lead, Epicardial pacing lead

## Abstract

Epicardial pacing lead fixation is employed in patients with cavopulmonary anastamosis (Glenn shunts) when they need permanent pacing. Epicardial pacing in these patients may malfunction due to high pacing thresholds or diaphragmatic pacing. A novel technique of transatrial insertion of two endocardial screw-in pacing leads through right anterolateral minithoracotomy could achieve synchronous atrioventricular pacing in a patient with Ebsteins anomaly with symptomatic sinoatrial and atrioventricular nodal disease.

## Case Report

**Figure 1:**
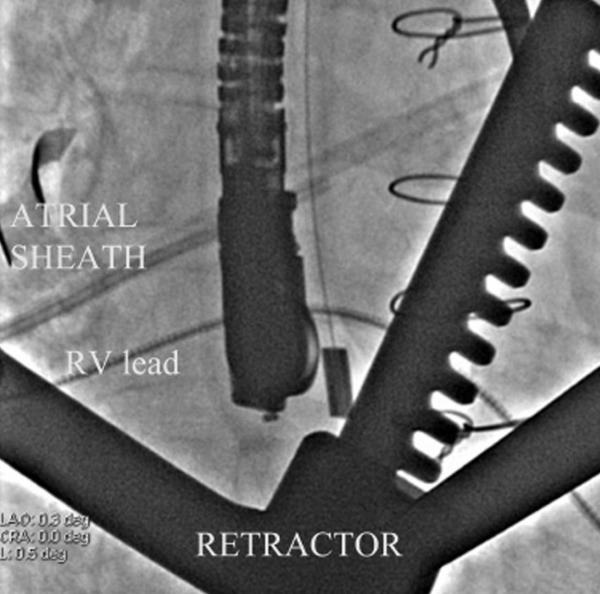
Figure 1 shows frontal plane fluoroscopy with retractors through the right anterolateral thoracotomy, transesophageal echocardiographic probe (TEE) guides placement of atrial and ventricular (RV) screw-in leads.

**Figure 2:**
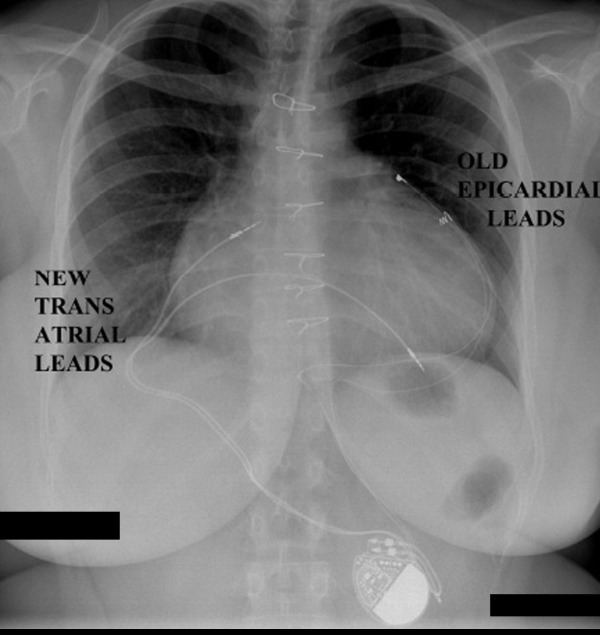
Figure 2 shows chest X-ray demonstration of both the old epicardial dual pacing leads and new transatrial endocardial screw-in leads; the pulsegenerator is located in the subxiphoid epigastric subcutaneous pocket.

A 23-year-old female with Ebstein's anomaly of tricuspid valve underwent one and half ventricular repair with tricuspid valve annuloplasty, atrial septal defect closure and cavopulmonary anastamosis of right superior venacava to the right pulmonary artery. She also needed dual chamber pacing for symptomatic bradycardia secondary to sinus nodal and atrioventricular nodal disease. There was no pre-excitation or tachyarrhythmias. Left atrial and left ventricular epicardial leads were connected to a pulse generator that was implanted in epigastric subcutaneous pocket. There was synchronous atrioventricular pacing with acceptable pacing thresholds during the time of implantation. Three months later, new onset symptomatic diaphragmatic pacing was observed from atrial lead. So the pacemaker was reprogrammed to pace ventricle alone. After another two months, diaphragmatic pacing was observed from ventricular lead too. The threshold voltage for ventricular capture was more than that of the diaphragm. The reasons for delayed onset diaphragmatic pacing through both leads almost five months after the epicardial lead placement and pacemaker implantation was not understood. Her symptoms related to bradycardia warranted permanent dual chamber pacing. Since extensive dissections will be needed through the previous surgical scars and adhesions in midline sternotomy and left thoracotomy, a second epicardial lead repositioning was not considered. A minimal incision right anterolateral thoracotomy was made and two introducer sheaths were inserted through two pursestring sutures placed on the right atrial free wall.(Figure 1) Two screw-in endocardial leads were inserted through the sheaths under transesophageal echocardiographic guidance into the right atrial appendage tip and right ventricular apex to achieve atrioventricular pacing. On a follow up of nine months, the patient was asymptomatic, pacing was synchronous and chest X-ray showed both the new and old leads. (Figure 2)

## Discussion

Epicardial pacing is employed in clinical situations associated with venous occlusions, cavopulmonary anastamosis and in small infants. The trend from epicardial towards endocardial lead placement in pacing has been steadily increasing over the years. Epicardial leads are associated with high thresholds resulting in need for higher pacing voltages. Inspite of anatomical placement of epicardial pacing leads away from the phrenic nerves, delayed epicardial lead related phrenic nerve stimulation and diaphragmatic pacing is reported. This may be related to scar syncytium formation over the left lateral surface of the epicardial surface of the heart leading to capture of both the myocardium and phrenic nerve. Peratrial insertion of endocardial leads could be used in situations where epicardial pacing leads to high thresholds and pacing failure or diaphragmatic pacing from phrenic nerve stimulation. There is also another advantage of minimizing growth related difficulties associated with endovenous leads in children. By using the transatrial approach one only has to consider the growth of the heart to calculate the additionally required length of the lead. Redundant lead loops or later revisions to reposition a lead by a few centimeters in order to prevent loss of capture due to growth, are of little concern in this access. This peratrial insertion of dual endocardial pacing leads offers a simpler solution to epicardial lead malfunction in patients with cavopulmonary anastamosis and other causes of venous occlusions.[1-4]
